# Complete genome sequence of *Weissella soli* strain 3-88 isolated from Kaldá river in Iceland

**DOI:** 10.1128/mra.00438-25

**Published:** 2025-07-23

**Authors:** Taya Tang, Søren Valsøe, Axel Soto-Serrano, Derek V. Byrne, Lukasz Krych, Jørgen J. Leisner

**Affiliations:** 1 College of Food Science and Engineering, Inner Mongolia Agricultural University 117454 , Hohhot, China; 2 Food Quality Perception and Society Team, Department of Food Science, Aarhus University, Aarhus, Denmark; 3 Department of Food Science, Faculty of Science, University of Copenhagen 4321, Frederiksberg, Denmark; 4 Department of Veterinary and Animal Sciences, Faculty of Health and Medical Sciences, University of Copenhagen 4321, Frederiksberg, Denmark; University of Maryland School of Medicine, Baltimore, Maryland, USA

**Keywords:** lactic acid bacteria, exopolysaccharide, heterosaccharide, flippase, *Weissella*

## Abstract

Here, we report the genome sequence of *Weissella soli* 3-88 isolated from Kaldá river in Iceland, using Oxford Nanopore sequencing. We classified the strain within *W. soli* by calculating and reporting its average nucleotide identity values compared to other *W. soli* strains.

## ANNOUNCEMENT

*Weissella soli*, found in soil and foods, has technological potential as a producer of exopolysaccharides (EPS) and lactic acid (1–3). A *W. soli* isolate was obtained from the Kaldá river in Iceland (Table 1) by incubating an unfiltered, undiluted surface water sample in all-purpose Tween (APT) broth (Difco, Sparks, MD) under microaerophilic conditions, using a candlelight, at uncontrolled room temperature for 7 days, before transfer to APT agar (1.35%; Merck, Darmstadt) and anaerobic incubation at 25°C. Gram-positive, catalase-negative lactic acid bacterial colonies were re-streaked and cultured in APT broth for genomic analysis.

**TABLE 1:** Sampling location description and genome characteristics for *Weissella soli* 3-88

Characteristic	Value
Description of location	
Location	Kaldá river, Iceland, near Eldborg crater
Time	30 August 2024
Type	River water
Geographic coordinates	64°46′43.2″N 22°18′06.1″W
Genome statistics	
Assembly size (bp)	1,678,388
Number of circular contigs	1
Contig N50 (bp)	1,678,388
Contig L50	1
GC (%)	43.9
Genome coverage	87 ×
Number of 5S rRNA	6
Number of 16S rRNA	6
Number of 23S rRNA	6
Number of tRNAs	65
Total number of genes	1678
Total number of CDS	1592
Total number of pseudogenes	7
TYGS taxonomy	*Weissella soli*
GTDB taxonomy	*Weissella soli*
EPS	*eps* cluster
Data accession	
BioProject	PRJNA1238846
BioSample	SAMN47485272
SRA	SRS24439780
Genome assembly	CP186084

Genomic DNA was extracted using the Bead-Beat Micro AX Gravity kit (A&A Biotechnology, Gdynia, Poland), which combined with the Native Barcoding Kit (SQK-NBD114.96, Oxford, UK), ensures isolation of high molecular weight DNA and generation of raw reads with an N50 of approximately 10 kb. DNA concentration was measured with a Qubit 4 fluorometer (Thermo Fisher, Waltham, USA). The final library with ligated motor protein was washed with Long Fragment Buffer to enrich for DNA fragments larger than 3 kb. DNA sequencing was conducted on a PromethION 2 Solo platform using R10.4.1 flow cell (FLO-PRO114M). Basecalling was conducted using the dna_r10.4.1_e8.2_400bps_sup@v5.0.0 basecalling model, and demultiplexing and adapter trimming were performed with Dorado v0.8.1 (https://github.com/nanoporetech/dorado). Bedtools v2.30.0 (4) and seqkit v2.1.0 (5) were utilized to convert the obtained bam files to FASTQ format and to remove duplicated reads, respectively.

Genome assembly and circularization were conducted using Hybracter v0.9.0 (6) using the flye --nano-hq model (7) and the Plassembler (8) plsdb_2023_11_03_v2 database. Reads were filtered at a minimum length of 1,000 bp and a quality score of 16, and the subsample depth value was set at 110×. After filtering, a total of 19,966 reads with an N50 of 9,799 bp were used for the assembly. Chromosome size was estimated automatically with the --auto flag. Polishing with medaka was omitted due to its potential to reduce accuracy (9). The genome was reoriented to start at DnaA using dnaapler v0.8.1 (10).

**Fig 1:**
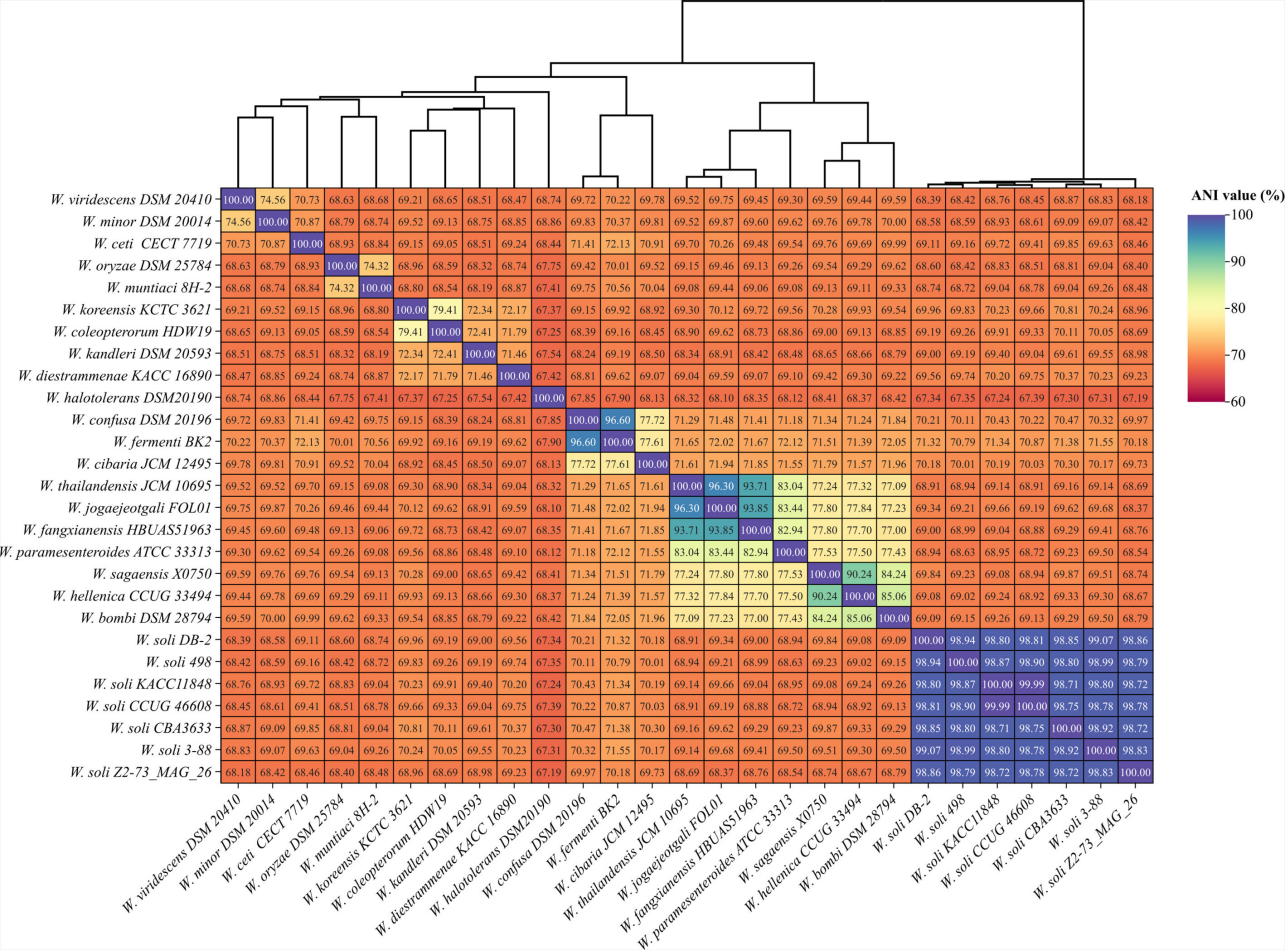
Heatmap of pairwise ANI values among *Weissella soli* strains and type strains of various *Weissella* species, including one freshwater isolate from this study and 26 strains obtained from GenBank. The dendrogram at the top illustrates hierarchical clustering of ANI values based on the Euclidean-distance method.

The complete genome was classified with the Type strain Genome Server (TYGS) v391 (11) and GTDBtk v. 2.3.2 against the Genome Taxonomy Database (release 214) (12). Annotation was conducted using the NCBI Prokaryotic Genome Annotation Pipeline (PGAP) (13–15). The average nucleotide identity (ANI) values between strain 3-88 and other *Weissella* spp. genomes were calculated by Orthologous ANI tool (16) and visualized using Chiplot (https://www.chiplot.online/) (Fig. 1).

The genome sequence contained genes (bp 1377492–1391637) corresponding to the *eps* cluster for EPS production via the Wzx-Wzy pathway, the most common pathway for heterosaccharide EPS biosynthesis in lactic acid bacteria, yet understudied in *Weisella* (17). The regulatory genes *cpsBC* were detected by homology with those of *Lactococcus lactis* KLDS 4.0325 using Protein Basic Local Alignment Tool (BLASTP) (18). The Wzx and Wzy flippase and polymerase functions were inferred based on transmembrane domain similarity to KLDS 4.0325, with domains predicted using DeepTMHMM v1.0 (19). Finally, BLASTP analysis resulted in no significant hits against the well-established glucansucrase of *Weisella confusa* LBAE C39-2 (18, 20).

## Data Availability

Raw sequence reads have been deposited at the NCBI under BioProject with accession PRJNA1238846, BioSamples with accession SAMN47485272, and SRA with accession SRS24439780. The assembled genome has been deposited at GenBank with accession number CP186084.
